# Endoscopic management of blue rubber bleb nevus syndrome: A case report

**DOI:** 10.3892/etm.2013.1303

**Published:** 2013-09-17

**Authors:** WEIWEI GUO, ZHAOYUAN PENG, XIAOWEI TANG, ZHIFEI ZHAO, ZHENG LIU

**Affiliations:** Medical Center for Digestive Diseases, Second Affiliated Hospital of Nanjing Medical University, Nanjing, Jiangsu 210011, P.R. China

**Keywords:** blue rubber bleb nevus syndrome, hemangioma, gastrointestinal hemorrhage, endoscopic management

## Abstract

Blue rubber bleb nevus syndrome (BRBNS) is a rare disorder characterized by multiple recurrent vascular malformations, including hemangioma, which primarily locate on the skin and gastrointestinal (GI) tract. The present study reports a 22-year-old female with iron-deficiency anemia and recurrent episodes of melena. The patient also exhibited characteristic venous malformations of the skin. Endoscopy revealed several hemangiomas in the GI tract. The hemangiomas were treated by ligation using a nylon cord, while small blue mucosal polypoid lesions were treated using a sclerosing agent during colonoscopy and double-balloon enteroscopy. The patient was reviewed regularly for three years following surgery and no further bleeding episodes were noted.

## Introduction

Blue rubber bleb nevus syndrome (BRBNS) is a rare disorder characterized by multiple recurrent vascular malformations, including hemangioma, which are primarily located on the skin and gastrointestinal (GI) tract (1). Other organ systems that may be affected include the central nervous system (2), liver, kidney, bladder, heart, thyroid and spleen. However, these are affected less often than the GI tract (3). Although cases that appear to have an autosomal dominant transmission have been reported (4,5), the majority of cases are sporadic with no family history of the disorder (6,7). Cutaneous angiomas are blue or purple, soft, rubbery, sessile, ‘nipple-like’ nodules with wrinkled and hyperhydrotic surfaces. However, they may also lie deep in the skin and appear as bluish macules (8).

A variety of therapeutic strategies have been proposed for the management of GI bleeding in BRBNS, including anti-angiogenic agents and surgical resection (9–18). However, no particular method has been demonstrated to be reliably effective in reducing bleeding or controlling blood loss permanently. Furthermore, surgical resection has been condemned as overly aggressive and unhelpful due to the theory that lesions may recur after removal (16). Endoscopy is considered a less invasive alternative for the treatment of BRBNS.

## Case report

**Figure 1.**
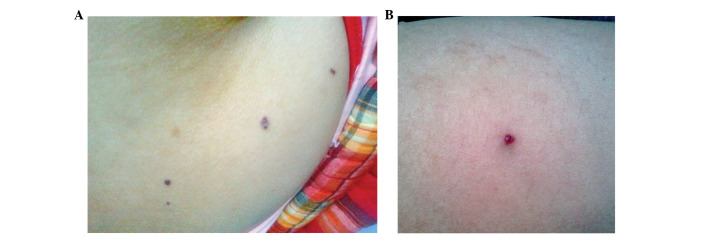
Venous malformations of the skin are deep-blue, soft, rubbery and easily compressible in (A) the hip and (B) the leg.

**Figure 2.**
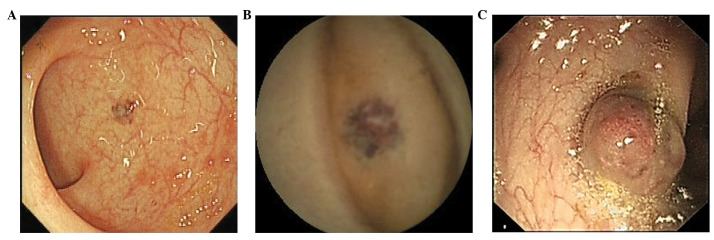
(A–C) Endoscopy revealed several mucosal polypoid lesions with abundant vasculature in the gastrointestinal (GI) tract. The lesions varied in size between 8 and 20 mm.

**Figure 3.**
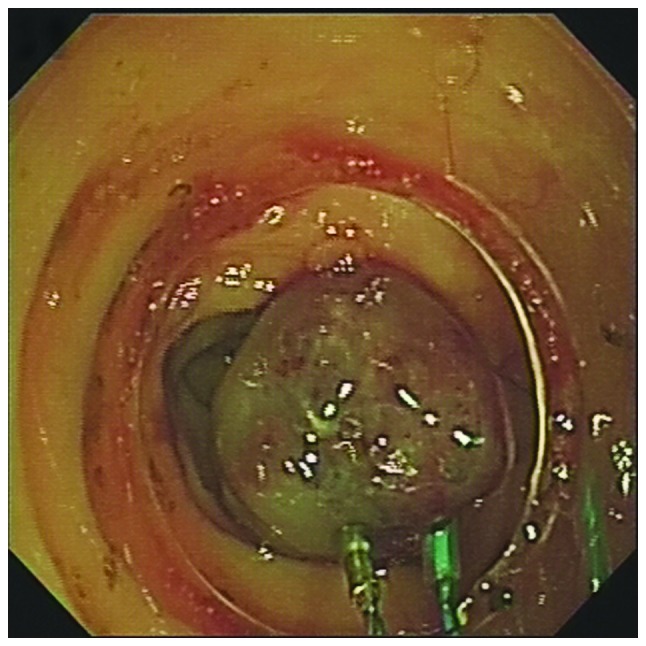
Two small blue mucosal polypoid lesions were treated with sclerotherapy and three hemangiomas were removed using a nylon cord under colonoscopy in the colon transversum.

The informed consent was obtained from the patient. In March 2012, a 22-year-old female with iron-deficiency anemia, secondary to recurrent episodes of melena, presented as an outpatient to the Department of Gastroenterology at the Second Affiliated Hospital of Nanjing Medical University (Nanjing, China). Since infancy, the patient had exhibited massive characteristic venous malformations of the skin, which were deep blue, soft, rubbery and easily compressible. Between 1991 and 2012, the patient had a history of recurrent GI bleeding and iron-deficiency anemia. The patient did not have any history of non-steroidal anti-inflammatory drug use or peptic ulcers. The patient had received several blood transfusions and, at 12 years of age, an abdominal surgery was performed to resect the hemangiomas in the stomach. A pathological report revealed hemangioma. According to laboratory examinations, the patient exhibited a hemoglobin level of 41.0 g/l, a red blood cell count of 2,410/mm^3^, a reduced white blood cell count and an increased neutrophil granulocyte count. Skin lesions of various sizes occurred on the abdomen, hip and leg of the patient (Fig. 1). The patient received a blood transfusion and hemostatic therapy. Following hemostatic therapy, a fecal occult blood test yielded negative results. In order to detect the origin of the hemorrhage, a colonoscopy and capsule endoscopy were performed. Endoscopy revealed several strawberry-like mucosal polypoid lesions with abundant vasculature in the colon and lesions ranging between 8 and 20 mm were blue-violet and sessile (Fig. 2). Colon lesions were removed in an attempt to eliminate bleeding under colonoscopy. During surgery, hemangiomas were ligated with a nylon cord and several small blue mucosal polypoid lesions were treated with sclerosing agent during colonoscopy (Fig. 3). The patient was discharged in a good condition. A repeat endoscopy at 3 months revealed completely normal mucosa in the original lesions. A number of small lesions that remained were treated with sclerosing agent. Over the subsequent three and a half years, the patient was reviewed regularly and no more bleeding episodes were noted.

We were able to diagnose the patient with BRBNS, according to the cutaneous angiomas and the GI mass lesions that were identified to be hemangiomas. However, it is necessary to differentiate BRBNS from hereditary hemorrhagic telangiectasia, Peutz-Jeghers syndrome, Klippel-Trénaunay syndrome and Maffucci syndrome.

## Discussion

BRBNS is a rare disorder characterized by multiple recurrent vascular malformations, including hemangiomas, which primarily locate on the skin and GI tract (1). Cutaneous angiomas are blue or purple, soft, rubbery, sessile, ‘nipple-like’ nodules with wrinkled and hyperhydrotic surfaces. However, they may also lie deep in the skin and appear as bluish macules. A number of studies have reported that BRBNS patients experience a gradual increase in pain (19). Pain may be caused by the contraction of smooth muscle fibers surrounding the vascular tumors (20). The molecular mechanisms underlying this disease are yet to be fully elucidated. It has been identified that normal endothelial cells of adult vessels do not show c-kit expression, whereas at least partial c-kit positivity has been reported in angiosarcomas (21). In addition, it has been demonstrated that pharmacological inhibition of the c-kit signaling pathway in cavernous hemangiomas by selective kinase inhibitors may offer options in the treatment of BRBNS patients (22). Nobuhara *et al* (23) identified mutations in the TIE2 gene that encode an endothelial cell tyrosine kinase receptor, which may govern the thickness of the smooth muscle wall of a vessel.

Various treatments for BRBNS are shown in Table I. Progress in endoscopic technology has advanced medical practice concerning the GI tract. Endoscopic diagnosis and treatment of conditions has now supplanted a number of surgical procedures and ongoing technical improvements, and innovations continue to extend the potential for endoscopic therapies (24). In the present case, the patient had exhibited gastrointestinal hemorrhage for 22 years. The patient had received several blood transfusions and iron replacement therapy for anemia by mouth. Endoscopy demonstrated that there were a number of hemangiomas in the GI tract. Further endoscopy was then undertaken to treat the hemangiomas using sclerosis and banding ligation. During a 12-month follow-up, the patient did not exhibit hemorrhage or anemia, and the hemoglobin level was in the normal range.

Surgery is an alternative therapy option. It is an effective method of hemostasis and allows the removal of hemangiomas simultaneously. However, if there are several vascular malformations along the whole digestive tract, surgical methods may not be feasible. Place (16) reported that multiple resectional surgeries (partial gastrectomy, partial small bowel resection, total abdominal colectomy and end ileostomy) resulted in significant long-term complications, including iron-deficiency anemia, nephrolithiasis, major depression and malnutrition. In the present case, endoscopic banding ligation and sclerotherapy were selected. Combined with interferon α, GI bleeding in the patient may be controlled effectively. The results achieved were comparable with those of surgery. Therefore, patients are more willing to accept endoscopic treatment than surgical therapy. Endoscopic management of BRBNS may not only increase the quality of life, but may also reduce the medical cost and hospitalization time of patients.

## Figures and Tables

**Table I.** Treatments for blue rubber bleb nevus syndrome.

Year of publication	Author	Therapeutic methods	Complications related to treatment	Outcome	Ref
1990	Maunoury *et al*	Nd:YAG laser and bipolar electrocoagulation	None	Avoided hemorrhagic recurrence of lesions in the small bowel	(10)
1996	Carr *et al*	Enterotomies	-	Stable at follow-up 5 years later	(14)
1999	Sala Felis *et al*	Endoscopic treatment by sclerosis and banding ligation	None	Effective	(9)
2001	Place	Multiple resectional surgeries (partial gastrectomy, partial small bowel resection, total abdominal colectomy and end ileostomy)	Iron-deficiency, anemia nephrolithiasis, major depression, and malnutrition	Significant long-term complications	(16)
2003	Ng and Kong	Argon plasma coagulation	-	Simple, inexpensive and effective treatment	(13)
2006	Anzinger *et al*	Therapeutic double balloon enteroscopy	None	Effective	(11)
2007	Okabayashi *et al*	Laparoscopic surgery	None	Without iron deficiency anemia for a year following the operation	(12)
2008	Emami *et al*	Endoscopic polypectomy resection	None	Useful	(18)
2010	Blaise *et al*	Polidocanol foam sclerotherapy	None	Technique has not yet been standardized	(15)
2012	Yuksekkaya *et al*	Sirolimus	No drug adverse reaction at 20-month follow-up	Vascular masses were reduced rapidly and there was no gastro-intestinal bleeding	(17)
